# Supplementary material for: Influence of agility training on body-size and object solidity perception in pet dogs
Source: PLoS One. 2026 Jan 7;21(1):e0338647. doi: 10.1371/journal.pone.0338647 (PMC12779069; doi:10.1371/journal.pone.0338647)
Supplement: S1 Table — (DOCX) [file pone.0338647.s001.docx]

Table S1. p-values adjusted for multiple comparison using the Bonferroni method.

| Combination | Training | Adjusted p-value | Probability | LCL | UCL |
| --- | --- | --- | --- | --- | --- |
| 11 | Trained | <0.01* | 96% | 0.796 | 0.999 |
|  | Untrained | <0.01* | 100% | 0.839 | 1.000 |
| 12 | Trained | <0.01* | 88% | 0.688 | 0.975 |
|  | Untrained | <0.01* | 91.7% | 0.730 | 0.999 |
| 13 | Trained | **0.1346** | 72% | 0.506 | 0.879 |
|  | Untrained | **0.3463** | 54.5% | 0.322 | 0.756 |
| 14 | Trained | <0.01* | 91.7% | 0.730 | 0.990 |
|  | Untrained | <0.01* | 100% | 0.860 | 1.000 |
| 15 | Trained | **1** | 56% | 0.349 | 0.756 |
|  | Untrained | **1** | 69.6% | 0.370 | 0.870 |
| 16 | Trained | <0.01* | 100% | 0.863 | 1.000 |
|  | Untrained | <0.01* | 100% | 0.852 | 1.000 |
| 17 | Trained | <0.01* | 100% | 0.846 | 1.000 |
|  | Untrained | <0.01* | 95.2% | 0.762 | 0.999 |
| 18 | Trained | <0.01* | 100% | 0.858 | 1.000 |
|  | Untrained | <0.01* | 100% | 0.850 | 1.000 |
