# Supplementary figures and images for: Influence of agility training on body-size and object solidity perception in pet dogs
Source: PLoS One. 2026 Jan 7;21(1):e0338647. doi: 10.1371/journal.pone.0338647 (PMC12779069; doi:10.1371/journal.pone.0338647)

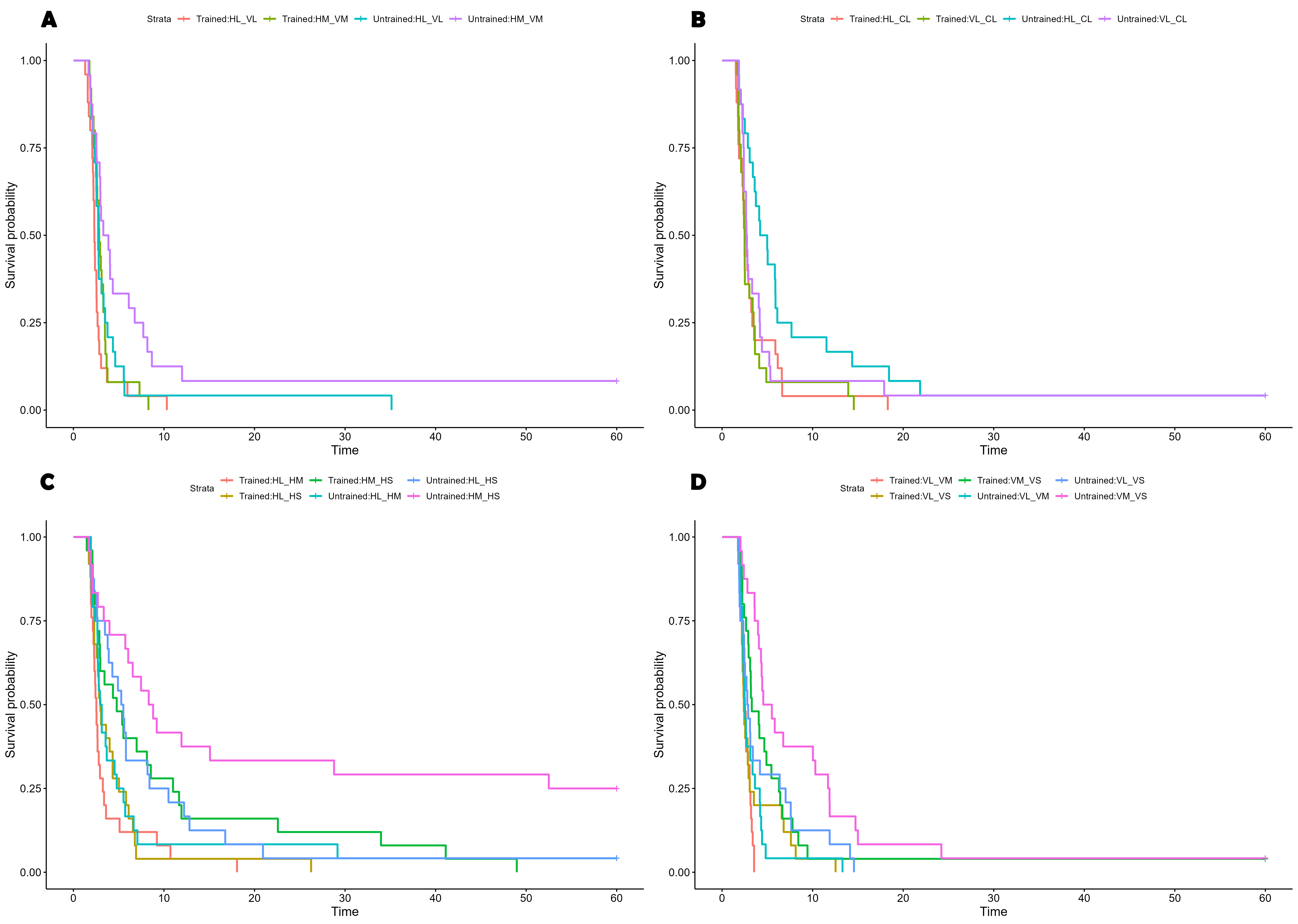

Supplement: S1 Fig — First letter of the names in the legend indicate shape (H-horizontal, V-vertical, C-circular) and the second indicates size (L-large, M-medium, or S-small). (PNG) [file pone.0338647.s002.png]

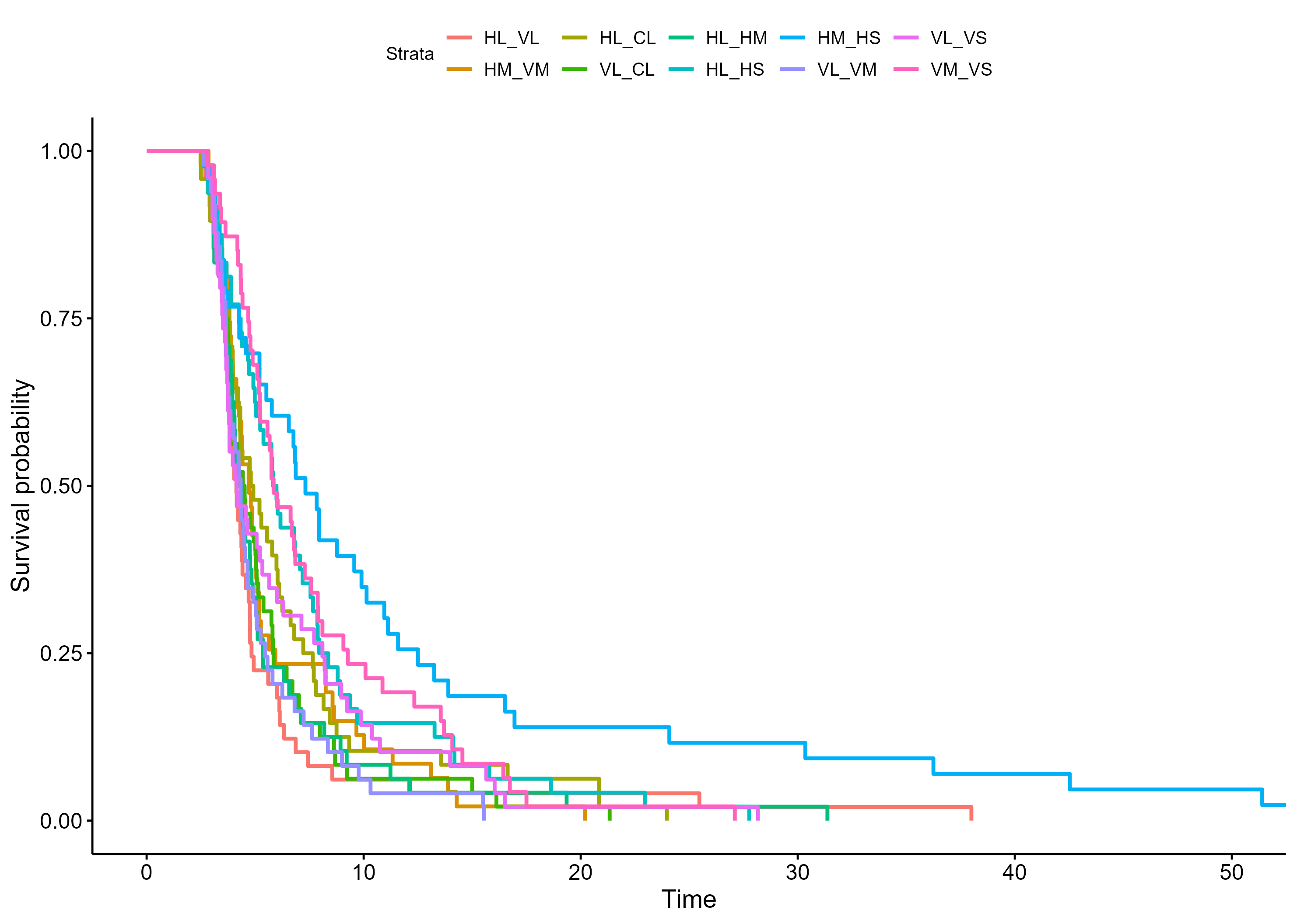

Supplement: S2 Fig — (PNG) [file pone.0338647.s003.png]

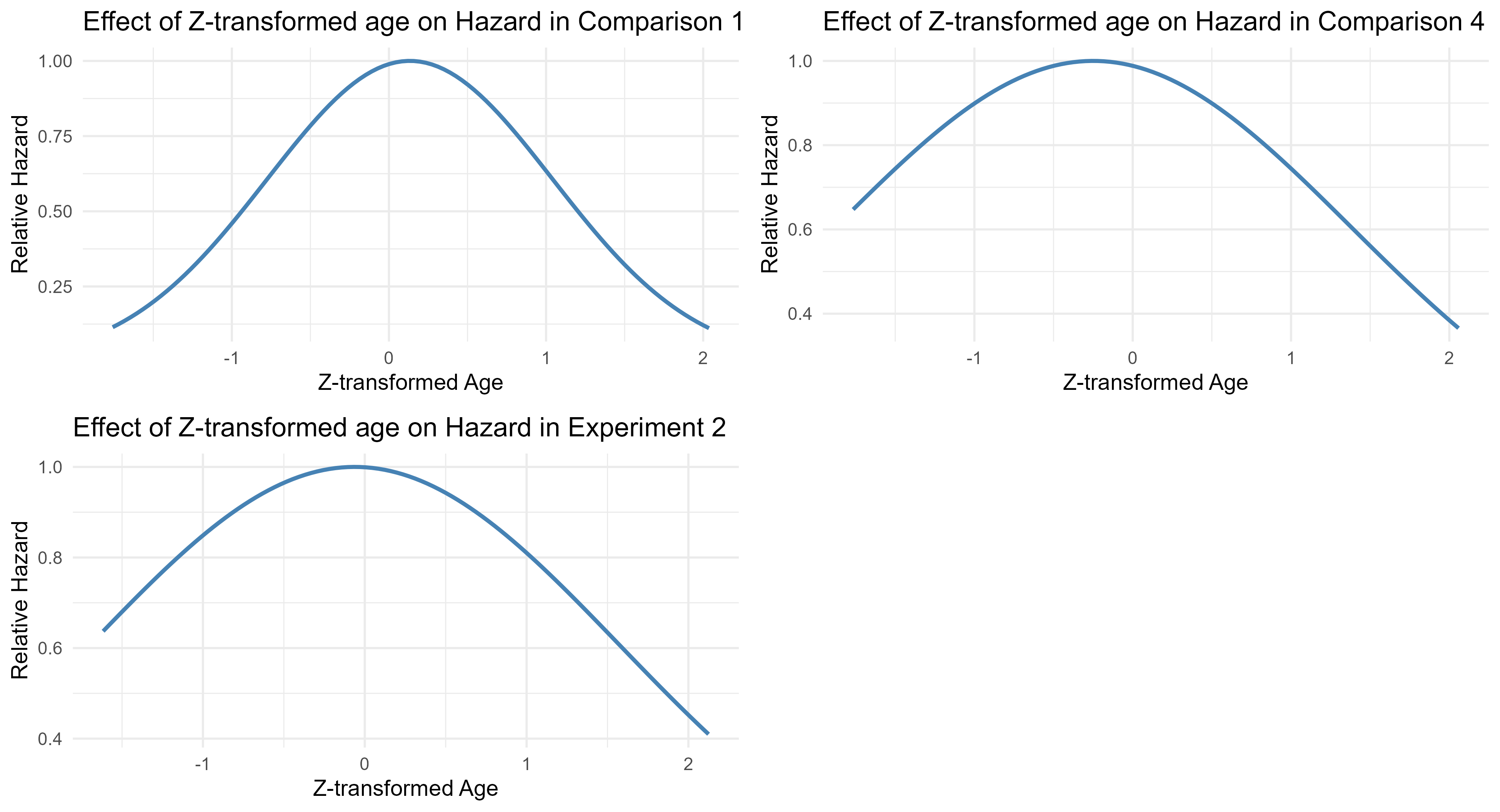

Supplement: S3 Fig — The names in the legend are first two letters of the material covering the material (Pl is short for plastic plant, Pa for paper, and Wo for wood). (PNG) [file pone.0338647.s004.png]
