# Supplementary material for: Influence of agility training on body-size and object solidity perception in pet dogs
Source: PLoS One. 2026 Jan 7;21(1):e0338647. doi: 10.1371/journal.pone.0338647 (PMC12779069; doi:10.1371/journal.pone.0338647)
Supplement: Attachment — Submitted filename: PONE-D-25-25871_Reviewer Comments.pdf [file pone.0338647.s010.pdf]

Manuscript Number: PONE-D-25-25871

The authors present an important and timely study on body awareness in dogs. While previous research has attempted to explore this aspect of canine cognition, the authors provide clearer evidence by employing a two-choice paradigm and testing dogs with varying levels of training experience. This study is likely to make a meaningful contribution to the field of animal cognition, particularly in understanding self-awareness. However, I have several questions and suggestions that I believe could help strengthen and clarify the manuscript.

Major Comments

The title should be reconsidered to avoid potential misunderstanding. While “body-size perception” is an appropriate and well-established term, the use of “body-shape perception” and “body-solidity perception” may be problematic. To the best of my knowledge, the term “body-shape perception” is not commonly used in animal cognition research, including previous studies on body awareness. Moreover, the distinction between body size and body shape appears ambiguous and potentially redundant, as the two concepts may substantially overlap in meaning. Furthermore, “body-solidity perception” is inaccurate, as the subjects were perceiving the solidity of the covering materials, not their own bodies.

In the analysis, all combinations of windows were treated equally; however, some should be interpreted separately. There are two types of combinations: one where both doors are available (e.g., the Horizontal large enough and the Vertical medium size), and another where one side is unavailable (e.g., the Horizontal large enough and the Vertical too small). While it is reasonable to define the larger and the particular shape as the correct choice, it is problematic to analyze both types of conditions in the same way. In the former case, the “incorrect” option is still “available,” whereas in the latter case, the “incorrect” option is actually “unavailable.” This difference affects how the dogs’ choices should be interpreted.

In the Results section, it would be helpful to include a comparison between the two-door choices as well as between the trained and untrained groups. While p-values of the former comparisons are reported, those for the latter are not. A comparison between the training conditions is essential for clarifying how prior experience contributes to the development of body awareness.

Reporting the exact choice ratios would enhance the interpretability of the results, rather than relying solely on p-values. I also recommend presenting some of the findings as figures to

improve clarity, as too many tables can make the data difficult to follow. Additionally, the current table format does not clearly indicate which door was chosen in each trial, making it difficult for readers to quickly grasp the key outcomes.

To support the claim that animals possess self-awareness, some evidence of self-directed behavior is generally required (Gallup & Anderson 2018, *Behavioural Processes*; Gallup & Anderson 2020, *Psychology of Consciousness: Theory, Research, and Practice.*; Kakrada & Colombo 2022, *Learning and Motivation*). Self-directed behavior refers to actions in which animals inspect or interact with their own body, such as mirror-guided self-exploration (Gallup, 1977, *American Psychologist*). If any potentially relevant behaviors were observed—such as hesitation or re-examination of body size before attempting to pass through a narrow opening—I suggest mentioning them, even if only as qualitative observations.

Miner Comments

Title

Line 1: As noted in the Major Comments, “body-shape perception” should be incorporated into “body-size perception,” as the distinction between the two is unclear. Additionally, “body-solidity perception” is an inaccurate term, as it refers to the perception of the covering materials rather than the subjects’ own body.

Line 2: The term “pet” is likely unnecessary, as the majority of studies involving dogs already use pet dogs as subjects. Unless there is a specific reason to distinguish them from working or shelter dogs, the term can be omitted to improve conciseness.

Abstract

Line 28: It is unclear what exactly became “faster” or “slower.” Specifying the exact parameter being described would improve the clarity of the results.

Introduction

Line 70: This paragraph and the one that follows could be condensed and integrated to reduce redundancy.

Line 81: The citation style appears to be inconsistent with the journal’s guidelines.

Line 83: A citation is needed to support this statement.

Line 108: If the final “building block” has been demonstrated, is it still necessary to empirically test the two preceding ones?

Ethical statement

Line 167: How much time did each subject spend participating in the experiment?

Materials and methods

Line 231: What was the inter-trial interval used in the experiment?

Line 233: How long was the “short break” mentioned in the procedure?

Line 239: Does this definition of “first approach” follow any precedent in previous research? If this criterion was developed specifically for this study, it would be important to provide supporting evidence or justification to ensure its validity.

Line 242: Why was latency not measured from the moment the owner unleashed the dog? Measuring latency from that point could provide insight into whether the subject approached the door immediately or hesitated before making a decision—an important distinction that can help detect behaviors that may indicate self-directed processing.

Line 253: Why was the rationale for including the z-transformed trial number as a variable in Experiment 2?

Experiment 1: Table 1: In the “Vertical” column, the label “C” should be corrected to “CW” for accuracy.

Experiment 1: Results

Line 330: Does the term “training” refer to agility training specifically, or to the training phase within the experimental procedure?

Line 359: This combination should not be interpreted in the same way as the others, as the subjects can available both windows in this case, unlike in the other two combinations.

Line 363: The interpretive value of this measurement may be limited, as the subjects are capable of passing through both “Large” and “Medium” windows. If both options are equally accessible, it becomes difficult to determine whether the subjects’ choices reflect body-size

awareness or are simply arbitrary.

Experiment 1: Discussion

Paragraph 3, line 5: To more clearly assess this possibility, a combination of the Circular window with either the Horizontal or Vertical too small should be tested. These options appeared to be less preferred by the subjects.

Experiment 2: Object solidity

Subjects: How long was the interval between Experiment 1 and Experiment 2 for subjects that participated in both experiments?

Experiment 2: Results

Paragraph 2, line 10: Should the final combination be 1 and 3 rather than 1 and 2?

Experiment 2: Discussion

Paragraph 1, line 5: Are the relevant statistical results reported in the Results section? If not, please consider including them to support the interpretation of the findings and ensure consistency with the hypotheses or claims made in the Discussion.

Paragraph 2, line 3: Does agility training include any components that involve learning about material solidity?

Paragraph 2, line 10: Providing details of a representative agility training program would help readers better understand and contextualize the results. I strongly recommend including this information either in the main manuscript or as supplementary material.

General Discussion

Paragraph 1, line 1: In the case of dogs, the olfactory self-recognition test has been developed as a means to investigate self-awareness (Horowitz 2017, *Behavioural Processes*). This study may be worth citing, as it provides an alternative approach to assessing self-awareness in dogs. Together with the accompanying comment article, it offers valuable insights into how self-awareness in animals can be examined (Gallup & Anderson 2018, *Behavioural Processes*).

Paragraph 3, line 12: It would be helpful to clarify the relationship between the “first approach” and the “first attempt.” What distinguishes trials in which subjects attempted to go through

the same window they first approached from those which they did not? How many times do subjects approach the windows before making their “first attempt”? Are all subjects “walked through” the window on their “first attempt”?

Paragraph 4, line 8: The logical link between this idea and the preceding statement is unclear. Please consider providing additional explanation to clarify the relationship.

Table S1: The raw p-values should be omitted; only the adjusted p-values should be reported.

References: The published month and date may be unnecessary. The title should follow sentence case, capitalizing only the first word and any proper nouns. Scientific names should be italicized.
