# Supplementary material for: Influence of agility training on body-size and object solidity perception in pet dogs
Source: PLoS One. 2026 Jan 7;21(1):e0338647. doi: 10.1371/journal.pone.0338647 (PMC12779069; doi:10.1371/journal.pone.0338647)
Supplement: Attachment — Submitted filename: PONE-D-25-25871_Reviewer Comments#2.pdf [file pone.0338647.s012.pdf]

Reviewer #1: Manuscript Number: PONE-D-25-25871

The manuscript has been significantly improved, and I appreciate the authors' thorough and thoughtful responses to my previous comments. I believe the paper has been sufficiently revised; however, a few minor issues remain, primarily related to formatting and consistency.

Several points regarding the formatting of numerical values and the citation of figures and tables could be improved for clarity and consistency throughout the manuscript.

1. Add spaces between numbers and units or symbols. For example, revise "6mx7m" to "6 m × 7 m."

2. Capitalize the initial letter when referring to figures and tables.

3. Ensure consistency in reporting p-values. Since very small p-values are reported as $p < 0.01$, all other p-values should be presented with two decimal places.

4. Several p-values below 0.01 are shown as exact values. For consistency, these should also be reported as $p < 0.01$, unless there is a specific reason to report the exact value.

The following line numbers refer to the Revised Manuscript with Track Changes.

Line 47-70: It is somewhat unnatural that only "the first building block" is separated from the others. I recommend combining these two paragraphs into a single unified paragraph, starting a new one from Line 60 onward. Alternatively, consider separating each building block into its own individual paragraph.

Figure 3-7, 9: The title of the rightmost graph should be corrected to "Opening."

Line 485: The influence of the agility training should be mentioned here.

Line 548: This statement appears to contradict the discussion in Line 539. Since the following sentence states that the exact reason is unclear, I suggest deleting this sentence for clarity and consistency.

Line 552: The prediction presented here seems unnecessary and could be removed to keep

the discussion more focused and concise.

Table 9: As in Table 2, include the combination number for clarity.

Line 660: Add the appropriate table number.

Line 676: The table number should be corrected to “Table 11.”

Line 683: The influence of the agility training should be mentioned here as well.

Line 828: The phrase “Phylogenetic placement” seems more appropriate than “cognitive abilities” in this context.

Line 885: The figure number should be corrected to “Figure S2.”

Line 893: The figure legend here is duplicated from Line 885 and should be removed.
