# Supplementary material for: Influence of agility training on body-size and object solidity perception in pet dogs
Source: PLoS One. 2026 Jan 7;21(1):e0338647. doi: 10.1371/journal.pone.0338647 (PMC12779069; doi:10.1371/journal.pone.0338647)
Supplement: Attachment — Submitted filename: Response to Reviewers.docx [file pone.0338647.s011.docx]

Reviewer #1: Manuscript Number: PONE-D-25-25871

The authors present an important and timely study on body awareness in dogs. While previous research has attempted to explore this aspect of canine cognition, the authors provide clearer evidence by employing a two-choice paradigm and testing dogs with varying levels of training experience. This study is likely to make a meaningful contribution to the field of animal cognition, particularly in understanding self-awareness. However, I have several questions and suggestions that I believe could help strengthen and clarify the manuscript.

Major Comments
The title should be reconsidered to avoid potential misunderstanding. While “body-size perception” is an appropriate and well-established term, the use of “body-shape perception” and “body-solidity perception” may be problematic. To the best of my knowledge, the term “body-shape perception” is not commonly used in animal cognition research, including previous studies on body awareness. Moreover, the distinction between body size and body shape appears ambiguous and potentially redundant, as the two concepts may substantially overlap in meaning. Furthermore, “body-solidity perception” is inaccurate, as the subjects were perceiving the solidity of the covering materials, not their own bodies.

**Our response**: the title has been changed to: Influence of agility training on body-size and object solidity perception in pet dogs

In the analysis, all combinations of windows were treated equally; however, some should be interpreted separately. There are two types of combinations: one where both doors are available (e.g., the Horizontal large enough and the Vertical medium size), and another where one side is unavailable (e.g., the Horizontal large enough and the Vertical too small). While it is reasonable to define the larger and the particular shape as the correct choice, it is problematic to analyze both types of conditions in the same way. In the former case, the “incorrect” option is still “available,” whereas in the latter case, the “incorrect” option is actually “unavailable.” This difference affects how the dogs’ choices should be interpreted.

**Our response**: In our analysis, we tested if the animals had a preference for one side or the other, i.e. do animals pick one opening significantly above chance when two openings were presented. Importantly we looked at the first side approached and first side attempted – so before they actually experienced that one choice was not really available after all (because they did not fit through it). To be able to do so, we defined one opening as “correct” (coded as 1) and the other as “incorrect” (coded as 0), which matches the predictions of our hypotheses. In each model, we included the effect of the “combination number” (i.e. the actual combination presented), which tells us if there is a difference between each combination. Therefore, we did test for this specific difference between the two types of combinations presented when analysing which opening they first approached and attempted. For the actual opening they walked through, we only analysed the combinations where both options were passable.


In the Results section, it would be helpful to include a comparison between the two-door choices as well as between the trained and untrained groups. While p-values of the former comparisons are reported, those for the latter are not. A comparison between the training conditions is essential for clarifying how prior experience contributes to the development of body awareness.

**Our response**: We added the p-values in text where appropriate (in comparisons 3 and 4 of Experiment 1).


Reporting the exact choice ratios would enhance the interpretability of the results, rather than relying solely on p-values. I also recommend presenting some of the findings as figures to improve clarity, as too many tables can make the data difficult to follow. Additionally, the current table format does not clearly indicate which door was chosen in each trial, making it difficult for readers to quickly grasp the key outcomes.

**Our response:** We provided additional graphs in the result section to improve clarity. The graphs show the ratio of choices per training group per condition. The ratios were already reported as percentages of trials where they chose the predicted correct option in the tables along with the respective confidence intervals. The predicted correct choices are explained in the statistical analysis section and at the end of the introduction.

To support the claim that animals possess self-awareness, some evidence of self-directed behavior is generally required (Gallup & Anderson 2018, Behavioural Processes; Gallup & Anderson 2020, Psychology of Consciousness: Theory, Research, and Practice.; Kakrada & Colombo 2022, Learning and Motivation). Self-directed behavior refers to actions in which animals inspect or interact with their own body, such as mirror-guided self-exploration (Gallup, 1977, American Psychologist). If any potentially relevant behaviors were observed—such as hesitation or re-examination of body size before attempting to pass through a narrow opening—I suggest mentioning them, even if only as qualitative observations.

**Our response:** We did not observe any self-directed behaviours that would be worth mentioning. However, as mentioned in the introduction, we focused on a very small aspect of self-awareness, which should be achievable by any animal with active locomotion. This aspect of self-awareness probably does not require self-directed behaviours and thus could be achieved simply from sensory information gathered through interaction with the environment. We did not want to claim with this study that animals possess self-awareness but rather focus on perception of physical attributes.


Miner Comments
Title
Line 1: As noted in the Major Comments, “body-shape perception” should be incorporated into “body-size perception,” as the distinction between the two is unclear. Additionally, “body-solidity perception” is an inaccurate term, as it refers to the perception of the covering materials rather than the subjects’ own body.

**Our response:** We changed the title accordingly. We also replaced any mention of body-solidity perception with object solidity perception. We would still prefer to keep the term shape perception. Although shape is technically incorporated in size perception, as shape is just size in different directions, it was not explicitly tested previously if animals perceive these measurements differently. This distinction between size and shape also helps separate different conditions.

Line 2: The term “pet” is likely unnecessary, as the majority of studies involving dogs already use pet dogs as subjects. Unless there is a specific reason to distinguish them from working or shelter dogs, the term can be omitted to improve conciseness.

**Our response:** Our lab often works with pet dogs, free-ranging dogs, and dogs raised in a wild park (pack dogs). Therefore, specifying that these are pet dogs may be important to some readers.

Abstract
Line 28: It is unclear what exactly became “faster” or “slower.” Specifying the exact parameter being described would improve the clarity of the results.

**Our response**: rephrased this line

Introduction
Line 70: This paragraph and the one that follows could be condensed and integrated to reduce redundancy.

**Our response**: the condensed paragraph now reads as follows:

To our knowledge only a few studies investigated the ability to put the **properties of one’s body in relation with the properties of other objects** in their environment ^[1–9]^. Awareness of one's **own body size** is the most commonly studied **property of body as an object**, and it is usually studied in the context of species whose males fight for resources (territory, potential mates, etc.) (e.g. ^[10,11]^). In that context, it is beneficial for an individual to have a perception of one’s own relative size to be able to assess how much bigger or smaller the opponent is. Only few studies so far tested body size perception in the context of foraging and moving through the environment ^[1–9]^. The paradigm most frequently used is presenting the animals with gaps of different sizes to go through or walk over. If the animals are aware of their body size, they should choose to walk through bigger gaps when available, adjust their bodies accordingly as the gaps get smaller, and be more hesitant to attempt going through small gaps. Alternatively, some studies used a single gap paradigm in which case the latencies to approach or reach the reward and the behavioural modifications (e.g. body pose, ducking the head, etc.) were taken as the measure of the awareness of body size. Animals across taxa seem to be aware of their body size and adjust their behaviour as they move through or walk over differently sized gaps ^[1–4,12]^, suggesting that understanding of one’s own size is indeed crucial for successful navigation through the environment. The only study that focused on other properties of body as an object (other than shape and size) was carried out on brown rats ^[5]^. The rats had to cross one of the three bridges presented in the enclosure to reach a reward, however only one bridge was fixated, while the other two were loose (acting like a see-saw). The brown rats seem to be able to put their own body weight in the relation to the solidity of the object they walk on. However, out of 25 animals, only 8 developed a probing behaviour (tapping the bridge with their paws) to discriminate between bridges, 12 gave up after a few falls, and the remaining 5 did not develop the correct strategy but kept on trying. This raises an interesting point that although the rats may be aware of their weight and how it interacts with a loose bridge, it does not mean that animals develop the correct strategy to test the properties of another object in the environment and, as mentioned by the authors, some rats might have perceived the falling off the bridge as too big of a risk to invest more energy to finding a solution.

Line 81: The citation style appears to be inconsistent with the journal’s guidelines.

**Our response**: corrected

Line 83: A citation is needed to support this statement.

**Our response**: the phrasing here was changed as we condensed the two paragraphs as mentioned in the point above. The needed references were added where appropriate. See the paragraph added in the response above.

Line 108: If the final “building block” has been demonstrated, is it still necessary to empirically test the two preceding ones?

**Our response**: Building blocks may differ between species as they may use different senses to experience them, so within species it would be important to define each of the building blocks to be able to explain how they develop. Therefore, it is hard to define how building blocks may relate to one another without testing for each of them separately. This is also in line with our results that shape and size may be assessed separately.

Ethical statement
Line 167: How much time did each subject spend participating in the experiment?

**Our response**: specified approximate duration of the experiment of 40 minutes (line 208*)

 *the lines specified here and throughout this document refer to revised manuscript with tracked changes

Materials and methods
Line 231: What was the inter-trial interval used in the experiment?

**Our response**: The inter-trial interval was the time needed for the experimenter to setup the next trial and answer any possible questions that the owner may ask after the trial (often asking if they are doing everything as instructed and how their dog is doing in the study). This usually took a few minutes (1-5 minutes). The next trial started as soon as possible.

Line 233: How long was the “short break” mentioned in the procedure?

**Our response**: duration specified in line 274

Line 239: Does this definition of “first approach” follow any precedent in previous research? If this criterion was developed specifically for this study, it would be important to provide supporting evidence or justification to ensure its validity.

**Our response**: in the study by Lenkei et al. (2019) and by Horrowitz at al. (2021) they also defined approach (but did not provide exact measurement of distance from the opening). In the studies by Khvatov et al, they defined the approach as the situation where the tip of the nose/beak of the animal is within 10 cms from the opening. We used a well-established measurement (body length) used in dog studies as a consistent measurement for each subject as different subjects may significantly vary in size(to name a few studies using this measurement ^[1–5]^).
Line 242: Why was latency not measured from the moment the owner unleashed the dog? Measuring latency from that point could provide insight into whether the subject approached the door immediately or hesitated before making a decision—an important distinction that can help detect behaviors that may indicate self-directed processing.

**Our response**: We added the analysis of latency to approach in the results section of both experiments as suggested (before latency to reach reward). We added the required definition in statistical analysis section(s).


Line 253: Why was the rationale for including the z-transformed trial number as a variable in Experiment 2?

**Our response**: In experiment 2 there were only 3 combinations and they were presented with 6 trials per combination. The effect of the trial number here provides us with the information about the performance over time. In Experiment 1 each trial was a unique combination randomized across session, meaning the trial number does not provide additional information. We added the rationale for z-transforming the trial number in the statistical analysis section (line 295).

Experiment 1: Table 1: In the “Vertical” column, the label “C” should be corrected to “CW” for accuracy.

**Our response**: corrected

Experiment 1: Results
Line 330: Does the term “training” refer to agility training specifically, or to the training phase within the experimental procedure?

**Our response**: the term “training” refers to agility training. The same term is mentioned in the statistical analysis (line 292). We added a clarification in the brackets that training as a variable refers to the two groups of dogs (agility trained or untrained) (line 292).

Line 359: This combination should not be interpreted in the same way as the others, as the subjects can available both windows in this case, unlike in the other two combinations.

Line 363: The interpretive value of this measurement may be limited, as the subjects are capable of passing through both “Large” and “Medium” windows. If both options are equally accessible, it becomes difficult to determine whether the subjects’ choices reflect body-size awareness or are simply arbitrary.

**Our response (including previous point)**: These points are covered by the second major point. Copy of our response there: In our analysis, we tested if the animals had a preference for one side or the other, i.e. do animals pick one opening significantly above chance when two openings were presented. Importantly we looked at the first side approached and first side attempted – so before they actually experienced that one choice was not really available after all (because they did not fit through it). To be able to do so, we defined one opening as “correct” (coded as 1) and the other as “incorrect” (coded as 0), which matches the predictions of our hypotheses. In each model, we included the effect of the “combination number” (i.e. the actual combination presented), which tells us if there is a difference between each combination. Therefore, we did test for this specific difference between the two types of combinations presented when analysing which opening they first approached and attempted. For the actual opening they walked through, we only analysed the combinations where both options were passable.

Experiment 1: Discussion
Paragraph 3, line 5: To more clearly assess this possibility, a combination of the Circular window with either the Horizontal or Vertical too small should be tested. These options appeared to be less preferred by the subjects.

**Our response**:
Implemented: lines 550-554 -“ The exact reason why the subjects avoided the circular opening remains unclear. It would therefore be beneficial if future studies also included combinations where circular option is presented with medium and too small vertical and horizontal openings. Although, given that our subjects avoided the circular opening, if it is presented with a too small opening it might be perceived as an impossible choice, which will probably results in subjects not making a choice at all.”.

Experiment 2: Object solidity
Subjects: How long was the interval between Experiment 1 and Experiment 2 for subjects that participated in both experiments?

**Our response**: Specified the interval between the experiments: Line 221: The time interval between experiments was between 4 and 46 days, depending on the owner availability.

Experiment 2: Results
Paragraph 2, line 10: Should the final combination be 1 and 3 rather than 1 and 2?

**Our response**: corrected

Experiment 2: Discussion
Paragraph 1, line 5: Are the relevant statistical results reported in the Results section? If not, please consider including them to support the interpretation of the findings and ensure consistency with the hypotheses or claims made in the Discussion.

**Our response**: The relevant results for the statements in paragraph 1 are reported in table 8.

Paragraph 2, line 3: Does agility training include any components that involve learning about material solidity?

**Our response**: Agility training does not involve learning about material solidity as the training focuses on avoiding obstacles at high speed rather than interaction with materials.

Paragraph 2, line 10: Providing details of a representative agility training program would help readers better understand and contextualize the results. I strongly recommend including this information either in the main manuscript or as supplementary material.

**Our response**: We added a brief description of agility training - line 139-151: “An agility training program for dogs incorporates a variety of obstacles to challenge their physical abilities, coordination, and responsiveness while keeping the sessions engaging and fun. In the main training phase obstacles like tunnels, weave poles, jumps, A-frames, dog walks, and seesaws are introduced. Tunnels are flexible tubes that dogs run through, starting with straight ones and progressing to curved ones. Depending on the size of the dogs, they have to adjust (crouch down) to fit through. Weave poles require the dog to zigzag through upright poles, while jumps, such as single-bar or tire jumps, can be adjusted in height based on the dog’s size and skill level. A-frames and dog walks help improve balance and confidence on elevated surfaces, while seesaws teach dogs to handle shifting surfaces. Positive reinforcement, such as treats, toys, or verbal praise, is essential to encourage the dog and maintain focus, while directional commands like "left," "right," or "jump" improve communication. Sessions should usually be fun, kept short to avoid fatigue, and performed 2–3 times per week, with adjustments based on the dog’s fitness level, age, and experience.”

General Discussion
Paragraph 1, line 1: In the case of dogs, the olfactory self-recognition test has been developed as a means to investigate self-awareness (Horowitz 2017, Behavioural Processes). This study may be worth citing, as it provides an alternative approach to assessing self-awareness in dogs. Together with the accompanying comment article, it offers valuable insights into how self-awareness in animals can be examined (Gallup & Anderson 2018, Behavioural Processes).

**Our response**: citations added

Paragraph 3, line 12: It would be helpful to clarify the relationship between the “first approach” and the “first attempt.” What distinguishes trials in which subjects attempted to go through the same window they first approached from those which they did not? How many times do subjects approach the windows before making their “first attempt”? Are all subjects “walked through” the window on their “first attempt”?

**Our response**: We added the “**Relation between approach and attempt, and attempt and choice”** section at the end of results of both experiments, describing how many times the animals attempted to walk through the barrier they first approached, and walked through the barrier they first attempted. The necessary information was also added in statistical analysis and discussion where appropriate.

Paragraph 4, line 8: The logical link between this idea and the preceding statement is unclear. Please consider providing additional explanation to clarify the relationship.

**Our response**: implemented

Table S1: The raw p-values should be omitted; only the adjusted p-values should be reported.

**Our response**: implemented; changes not tracked by word

References: The published month and date may be unnecessary. The title should follow sentence case, capitalizing only the first word and any proper nouns. Scientific names should be italicized.

**Our response**: implemented; changes not tracked by word

Reviewer #2: In this study, two groups of dogs (untrained pet dogs vs. agility-trained dogs) were compared in a two-choice task requiring them to pass through one of two openings (Experiment 1: variation in size/shape; Experiment 2: variation in solidity). The authors conclude that: (i) dogs correctly assess their own body size; (ii) evidence regarding shape perception is less conclusive; (iii) dogs partially discriminate the solidity of obstacles; and (iv) experience (i.e., agility training) primarily affects decision speed and, to a lesser extent, shape perception.
This study offers a useful extension of the work by Lenkei et al. (2021), by testing multiple “building blocks” of the ecological self within the same species, and by introducing a two-option paradigm that allows for better control over motivational factors. The overall contribution is promising, but several major issues need clarification or improvement before publication.

- The categorization of dogs as “agility-trained” is based on relatively broad criteria (regular training and participation in A1-level competitions), which likely includes individuals with highly variable levels of intensity, frequency, and duration of training. To better characterize this group and assess its internal heterogeneity, it would be helpful to include additional information such as average weekly training time, age at training onset, and date of most recent competition participation.

**Our response**: We argue that this information provide accurate insight, since the weekly training time depends on the type of training/exercises covered that week and on the dog’s success. Therefore, more successful dogs may train less, which makes the interpretation of this information difficult. The main distinction is that all agility-trained dogs reached the A1-level and are active participants at agility dog competitions, while none of the untrained dogs did so. Furthermore, there is some heterogeneity in untrained dogs as well, as there are some dogs that train more or less than others. However, as mentioned in the discussion, we agree that it would be interesting to further investigate how different types or level of success influences body-awareness in dogs.

- The authors conclude that agility-trained dogs exhibit enhanced shape perception. However, this conclusion appears to overreach the empirical findings. The supporting results are limited to a single statistically significant condition, with a small effect size, and do not justify a clear claim of increased perceptual accuracy. The data more plausibly reflect faster decision-making or motor familiarity in specific configurations, rather than a finer or more conscious representation of body shape. It would be advisable to temper this conclusion in the discussion.

**Our response**: implemented; rephrased line 845 which now reads: “The agility training did not influence the shape and size perception. However, the agility training shortened the time needed for the dogs to make a decision when approaching the openings of different size and shape, but increased the time when approaching openings covered with different materials.”

- The analyses rely on an a priori categorization of choices as “correct” or “incorrect,” based on what the authors define as the optimal solution for the animal (e.g., a larger opening or a softer material). This approach carries the risk of imposing an anthropocentric decision framework, assuming that the dog’s perception of its own body necessarily aligns with human preferences or strategies in similar contexts. However, dogs’ individual preferences, contextual motivations, or sensory interpretations may diverge from this framework. It would be useful for the authors to acknowledge this limitation and to consider, in the discussion, the possibility that some choices judged as “suboptimal” from a human perspective may nonetheless reflect valid body representation from the animal’s point of view. A complementary approach could involve coding and analyzing bodily adjustments or hesitation behaviors as direct indicators of body perception, independently of the final choice.

**Our response**: The categorization of choices as correct and incorrect only serve a purpose in defining our variables (to be able to run a binomial model the way we did), but not in the interpretation of the results. Best example of this is that our finding that dogs had no preference between medium in large (which would be considered as incorrect by this categorization) in experiment 1 was not considered a failure, but rather we acknowledge that the openings could have been big enough and that our assumption of correct choice could have been wrong. We added the suggestion for future studies to also include behavioural coding in line 856.

- Given that one of the study’s stated aims is to evaluate the dog as a model for the systematic study of body and self-awareness (i.e., “ecological self”), the discussion would benefit from a more explicit comparative perspective involving other species. Specifically, previous studies using similar paradigms in elephants, rats, or birds should be mobilized not only qualitatively but also quantitatively, by citing observed effect sizes when available. This would help situate canine performance within a more rigorous comparative framework and strengthen the argument for their suitability as a model species. Such inclusion would also help clarify the potential species-specific contributions of ecological niche or life experience to the observed patterns.

**Our response**: we added a paragraph in the discussion to support the idea that dogs are a suitable model for the systematic study of body and self-awareness.

Reviewer #3: The manuscript presents novel data on the phenomenon of body-awareness in dogs. Although dogs have previously been the subject of similar research on multiple occasions, the authors' results provide valuable insights for advancing a comprehensive understanding of self-awareness across animal species.
Nevertheless, I have several critiques regarding the "Introduction," "General Discussion," and the validity justification of the experimental methodology.

Point 1. Lines 79–92: While the authors provide a general overview of methodology for studying body size awareness across animal species, the literature review is notably limited. The phenomenon of body size awareness—tested using body-as-obstacle tasks—has been documented in the following species (beyond those cited):

Budgerigars
Schiffner, I.; Vo, H.D.; Bhagavatula, P.S.; Srinivasan, M.V. Minding the gap: In-flight body awareness in birds. Front. Zool. 2014, 11, 64

Hooded crows
Khvatov, I.A.; Smirnova, A.A.; Samuleeva, M.V.; Ershov, E.V.; Buinitskaya, S.D.; Kharitonov, A.N. Hooded Crows (Corvus cornix) May Be Aware of Their Own Body Size. Front. Psychol. 2021, 12, 769397.

Ferrets
Khvatov, I.A.; Sokolov, A.Y.; Kharitonov, A.N. Ferrets (Mustela furo) Are Aware of Their Dimensions. Animals 2023, 13, 444.

Domestic cats
Pongrácz, P. Cats are (almost) liquid!—Cats selectively rely on body size awareness when negotiating short openings. iScience 2024, 27, 110799.

Wistar rats
Khvatov, I.A.; Ganza, P.N.; Kharitonov, A.N.; Samuleeva, M.V. Wistar Male Rats (Rattus norvegicus domestica) Are Aware of Their Dimensions. Animals 2024, 14, 3384.

Hermit crabs
Sonoda, K.; Moriyama, T.; Asakura, A.; Furuyama, N.; Gunji, Y.P. Can Hermit Crabs Perceive Affordance for Aperture Crossing? In Proceedings of the European Conference on Complex Systems 2012, Brussels, Belgium, 3–7 September 2012; Springer: Cham, Switzerland, 2013; pp. 553–557.

Krieger, J.; Hörnig, M.K.; Laidre, M.E. Shells as ‘extended architecture’: To escape isolation, social hermit crabs choose shells with the right external architecture. Anim. Cogn. 2020, 23, 1177–1187.

Recommendation for authors.
Integrate references to Schiffner et al. (2014), Khvatov et al. (2021, 2023, 2024), Pongrácz (2024), and Krieger et al. (2020). Highlight methodological parallels (e.g., aperture-crossing paradigms) to position this study within a broader comparative framework and enrich interpretation of your findings.

**Our response**: We added the missing references in the manuscript where appropriate.

Point 2. Lines 94–96. The authors claim that "the only study" investigating animals’ ability to relate bodily properties to environmental features was conducted on rats (assessing weight sensitivity). I disagree with this assertion.

While the cited rat study (14) explicitly demonstrated probing behavior to test substrate solidity, the capacity to integrate body properties with object features is inherently tested in any aperture-crossing paradigm—whether animals: decide to pass through a single opening, or select a viable opening among multiple options.
For example:
Birds folding wings mid-flight when traversing narrow vertical slits demonstrate pre-contact anticipation of collisions, directly relating body dimensions to aperture parameters before physical interaction (Schiffner et al., 2014).
Rats or ferrets immediately approaching and traversing a size-appropriate opening (when alternatives are impassable) similarly reveal this cognitive integration (Khvatov et al., 2023, 2024).

Recommendation for authors.
Revise to: "Few studies explicitly test animals’ ability to integrate bodily properties with object solidity. While rats (14) demonstrated substrate probing, aperture-crossing paradigms in birds and mammals inherently require this integration." Supporting citations: Schiffner et al. (2014), Khvatov et al. (2023, 2024), Pongrácz (2024).

**Our response**: This paragraph was condensed with the previous one and rephrased as a response to one of the previous point. It now reads: “To our knowledge the only a few studies investigated the ability to put the **properties of one’s body in relation with the properties of other objects** in their environment ^[1,10–17]^. Awareness of one's **own body size** is the most commonly studied **property of body as an object**, and it is usually studied in the context of species whose males fight for resources (territory, potential mates, etc.) (e.g. ^[6,18]^). In that context, it is beneficial for an individual to have a perception of one’s own relative size to be able to assess how much bigger or smaller the opponent is. Only few studies so far tested body size perception in the context of foraging and moving through the environment ^[1,10–17]^. The paradigm most frequently used is presenting the animals with gaps of different sizes to go through or walk over. If the animals are aware of their body size, they should choose to walk through bigger gaps when available, adjust their bodies accordingly as the gaps get smaller, and be more hesitant to attempt going through small gaps. Alternatively, some studies used a single gap paradigm in which case the latencies to approach or reach the reward and the behavioural modifications (e.g. body pose, ducking the head, etc.) were taken as the measure of the awareness of body size. Animals across taxa seem to be aware of their body size and adjust their behaviour as they move through or walk over differently sized gaps ^[1,5,10–12]^, suggesting that understanding of one’s own size is indeed crucial for successful navigation through the environment. The only study that focused on other properties of body as an object(other than shape and size) was carried out on brown rats ^[13]^. The rats had to cross one of the three bridges presented in the enclosure to reach a reward, however only one bridge was fixated, while the other two were loose (acting like a see-saw). The brown rats seem to be able to put their own body weight in the relation to the solidity of the object they walk on. However, out of 25 animals, only 8 developed a probing behaviour (tapping the bridge with their paws) to discriminate between bridges, 12 gave up after a few falls, and the remaining 5 did not develop the correct strategy but kept on trying. This raises an interesting point that although the rats may be aware of their weight and how it interacts with a loose bridge, it does not mean that animals develop the correct strategy to test the properties of another object in the environment and, as mentioned by the authors, some rats might have perceived the falling off the bridge as too big of a risk to invest more energy to finding a solution.”

Point 3. Lines 141–145. The authors argue that a two-opening paradigm is superior to a single-opening design. However, a critical question remains unaddressed: Why was a three-opening setup not employed? This alternative is methodologically justified for two primary reasons:

Ecological validity vs. cognitive load. Experiment 1 tested three aperture sizes; Experiment 2 used three barrier materials. A three-opening design would enable simultaneous presentation of all variants, granting subjects fuller choice—enhancing ecological validity. Counterpoint: three openings may overcomplicate the task, demanding higher attentional focus and potentially confounding results.
Methodological precedent. Three-opening paradigms have been successfully implemented in analogous studies (e.g., ferrets: Khvatov et al., 2023; rats: Khvatov et al., 2024), testing aperture shapes/sizes comparable to this work.
While I do not assert that three openings are inherently superior, the authors should justify their design choice relative to established methods. Results should also be contextualized against prior findings using multi-opening paradigms.

Recommendation for authors.
Explicitly state the rationale for selecting two openings. For example: "We retained the two-choice paradigm to: (a) Maintain comparability with prior canine body-awareness research (Lenkei et al., 2020); (b) Mitigate cognitive overload during extended 18-trial sessions. We acknowledge that while a three-opening design could enable direct comparison of all conditions, it risks reducing decision clarity. Future studies could test this adaptation."

**Our response**: Added an explanation why we decided against a three-choice paradigm. Lines 162-177:” Four other studies on snakes, rats, ferrets, and crows respectively ^[12–15]^ used a similar idea in a three-choice paradigm. This approach allows for simultaneous comparison of all three sizes (too small, medium, and large) presented in the study. However, there are several issues that arise from this approach. The main issue is the distance between the subject and the openings. The openings are either equidistant from the subject or from the reward, which makes one option more favourable. Indeed, three of those studies ^[13–15]^ reported a bias, as ferrets and crows preferred the central opening, while the rats avoided it. This issue alone makes this approach problematic, as we are no longer studying only the preference for the size or shape of the opening, but a combined preference for the location of the opening and the size and shape. Second issue is the number of combinations needed to test all of the possible combinations of openings presented. Finally, with the increase of combination there is an increase of “conditions” that have to be tested separately in the statistical analyses (either as different models or as different levels of a single variable). Accordingly, having three options would not allow us to tease apart which opening prompted the animal to make a specific choice and we would have to consider each opening as a separate effect that interacts with each other in a single trial. This would render the interpretation very difficult if not impossible.”

Point 4. Interpretation of Experiment 1 Results. Page 12. The authors claim: "Animals did correctly assess their size, as they approached, attempted, and went through the correct barrier above chance". This conclusion lacks sufficient empirical support. My rationale:

1. Behavioral sequence matters. Under high motivation, first-approach behavior is a stronger indicator of body size awareness than first-attempt or crossing choices. Animals may approach one aperture, investigate it, then cross through another (e.g., approach oversized → cross medium-sized). Critical metric: frequency of trials where the first-approached aperture was immediately crossed without prior investigation of alternatives. This is unreported.
2. Ambiguous first-approach data. Horizontal size effect: "Both groups approached the bigger option more often" (line 357) → Confirms preference for larger apertures, not selection of size-appropriate ones. Vertical size effect: Preference for large vs. small apertures only → No evidence dogs distinguished large vs. medium or medium vs. small.
3. Contrast with robust paradigms. Studies in ferrets/rats (Khvatov et al., 2023, 2024) used three-aperture designs: two impassably large openings and one passable small opening. Subjects preferentially approached and crossed passable apertures, proving size-appropriate selection—not just "bigger is better." Current data cannot confirm dogs chose passable over merely larger openings.

Recommendation for authors.
Reanalyze data to track:
1. First-approach-to-immediate-crossing sequences: How often did dogs cross the first aperture they approached?
2. Individual-level cases: Document trials where dogs approached oversized apertures but crossed medium-sized ones.

**Our response**: We added the analysis on how many times the subjects attempted to walk through the opening they first approached, and walking through the opening they first attempted.
Vertical size effect: we highlighted the interesting result where subjects approached at chance when small and large openings are presented, but still had a preference for large opening when approaching: line 510 “Interestingly, the subjects did not have a preference when approaching in combination 10 (vertical large vs small), but preferred the large opening when attempting to walk through. This is surprising, since we showed that in most cases subjects attempted the first opening approached. “
There are only two trials when a dog approached an oversized opening, but went through the medium-sized ones. Added to the discussion in line 737: “It is therefore possible that animals always approached the larger opening, but decided to investigate the second option and, as long as it was large enough (medium or large), they showed no preference to attempt or walk through. If this were the case, the first approach would be the most important measurement, while side first attempted and walked through would provide little information. In our data, there were only two trials when a subject approached a large opening, but walked through a medium sized one. Therefore, we can conclude that this is not the case and that side first attempted and walked through should also be taken into account”

Point 5. Demographic Variables in Statistical Models. The manuscript reports subjects' sex and age (with notably high age variance). However, the statistical analysis omits these variables as covariates in the models—a significant oversight requiring correction.

Recommendation for authors.
Refit all GLMM and COXME models including age (linear or log-transformed) and sex as covariates. Report whether the agility training effect persists after controlling for these demographic factors.

**Our response**: We disagree that sex or age should be included in the hypothesis. We had no hypotheses on the influence of sex or age, and it is not the focus of this study. Both age range and sex ratio (and averages) are similar in both of our test groups. We do not expect sex to have an effect on the performance as size and shape perception should not be sex dependent. Furthermore, all dogs were at least 14 months old, meaning they reached their maximum size and the mental image of their body should already be consolidated.

Point 6. Terminology: "Correct Choice" in Statistical Analysis (lines 307–312). The authors define a "correct choice" for each aperture combination in Experiment 1. This framing is methodologically unsound, particularly given that results contradicted initial hypotheses. Crucially:

1. Functional success ≠ predicted preference: A "correct" choice should reflect task success (reward attainment), not alignment with researcher predictions.
2. Example: Preferring a horizontal over vertical aperture is not "incorrect" if both yield rewards—it simply reflects subject preference.
3. Parallel issue in Experiment 2: The same problematic framing applies to barrier solidity choices.

Recommendation for authors.
Replace "correct choice" with "predicted choice" throughout Methods and Results. Explicitly state: "In combinations where both options were passable (e.g., horizontal vs. vertical), 'non-predicted' choices still enabled reward acquisition."

**Our response**:
We agree and understand how this can be confusing. Given our hypotheses, it is clearly defined what a correct choice means in each of the comparisons. This is what we tested in our statistical analysis. We believe it is clear to the reader what this entails. The terms “correct” and “incorrect” (coded 1 and 0) choice have to be defined for the statistical analysis. Although we agree that it is technically not an incorrect choice if animals received the reward, these labels here are functional (namely for the analysis) to serve as predictors for our hypothesis. We believe that the terms are not misleading the way they are described in the manuscript and that changing it in the manuscript might limit the clarity and/or reproducibility of the results as these terms correspond to the statistical analysis, and the full dataset and R script provided as supplementary material.

Point 7. Fatigue and Motivation Control in Experiment 2. Dogs encountered barriers requiring physical effort (tearing paper, pushing aside foliage) or presenting impassable obstacles (wood). The session structure—18 test trials plus 6 familiarization trials—imposes significant cognitive and physical load. This raises concerns about: progressive motivational decline; fatigue-induced performance decay (especially for latency and success probability within time limits). While breaks were mentioned, their effectiveness was not empirically verified.

Recommendation for authors.
1. Analyze trial-order effects: Segment sessions into thirds (early/mid/late) and compare key metrics (latency, success rate).
2. Include trial number as a covariate: Apply to all GLMM/COXME models in both experiments (currently partial for Exp2 only).
3. Acknowledge limitation: Discuss fatigue as a potential confounding factor in the "Limitations" section.

**Our response**: Statistical models for Experiment 2 already contain z transformed trial number as a continuous variable and we detected no effect of trial number as mentioned in the result section. Splitting it into thirds, as suggested, is not justified as it separates the data points into arbitrary groups, which may lead to false conclusions induced by this artificial division. Therefore, we believe this point has been covered by our analysis. The trial number was not included for Experiment 1 as there is no repetition of trials, which means that a certain trial number has a different effect for different subjects, which makes this effect difficult to interpret. The individual differences were already accounted for by including the subject ID as the random intercept. Therefore, we believe that it is not needed to include the trial number in models for Experiment 1.

Point 8. Interpretation of Circular Aperture Avoidance (Experiment 1). The authors hypothesize that avoidance of circular openings stems from "the necessity to jump with hind legs and the risk of hitting the brim." However, this interpretation lacks direct empirical support.

Recommendation for authors:
1. Reframe as a hypothesis: Explicitly state this explanation as one possible interpretation rather than a conclusion.
2. Conduct supplemental video analysis (if feasible): Quantify hind-leg jumping behavior during circular aperture crossings; Compare collision frequency (brim contact) between circular vs. other aperture shapes.
3. Acknowledge limitations: If video data is insufficient, explicitly note in "Limitations" that "the cause of circular aperture avoidance remains an unverified hypothesis."

**Our response**: It is already mentioned in the discussion that the “brim contact” is not visible from the video as it was not the focus of the study. Furthermore, adding a post-hoc hypothesis is against good science practice. It should be mentioned as the result/conclusion. We now explicitly stated that it is unclear why circular opening was avoided in both experiment 1 discussion and the general discussion. We added a suggestion that for future studies behavioural coding might be useful.

Point 9. Concern: Definition of "Medium" Apertures in Experiment 1. The authors define medium openings as "exactly the size the animal needed to walk through" (lines 272–273). However, this rigid definition overlooks critical biological and behavioral variability:
1. Individual differences in fur density/volume;
2. Posture-dependent body compression/expansion during passage;
3. Risk: Some "medium" apertures may have been functionally impassable or uncomfortable for certain dogs/postures, potentially explaining: absence of large vs. medium preference; observed aperture collisions.

Recommendation for authors:
1. Specify sizing protocol: Detail how "medium" was calculated (e.g., body measurement + X% clearance? Exact withers height?).
2. Discuss limitations: Address whether sizing accommodated: fur/coat volume; dynamic posture adjustments; inter-individual variability.
3. Acknowledge as potential confound: Cite this as a plausible explanation for null results in size comparisons.

**Our response**: We added that the measurements were rough and were taken using a measuring tape, for clarity. Although it is possible that some animals had it “easier” as their thicker fur may have caused the opening to be effectively bigger than it might have been for other subjects with shorter hair, by including the subject ID as the random intercept in the analysis we accounted for individual differences in the analysis.

Point 10. Comparison with Lenkei et al. (2021) on Aperture Shape. Lenkei et al. (the primary cited study) concluded that dogs (especially short-legged breeds) do not rely on shape perception, successfully traversing both horizontal and vertical slits through postural adaptation. By contrast, this study:
1. Expected preference for circular/vertical openings over horizontal ones;
2. Found no strong shape preference (beyond circular avoidance), except a trend toward horizontal openings in agility-trained dogs

Recommendation for authors.
Expand the General Discussion to reconcile these findings:
1. Emphasize that both studies demonstrate dogs' flexibility in navigating diverse shapes, but this work used a choice paradigm (vs. single-aperture trials in Lenkei et al.).
2. Note that the absence of shape preference (excluding circular) aligns with Lenkei et al.'s thesis of body-awareness plasticity.
3. Highlight the intriguing trend among agility dogs toward horizontal openings as task-specific behavior—potentially stemming from frequent tunnel/under-barrier training. Position this as a key question for future research.

**Our response**: We emphasized that both previous studies using similar setup demonstrated flexibility when navigating openings of different size and shape- line 784 “Although both previous studies demonstrated dogs’ flexibility when navigating openings of different size (and shape^[1]^), the previous studies never directly compared two openings of different size and shape.”

We rephrased the paragraph comparing our results with the results from lenkei et al, also mentioning the interesting finding that agility dogs preferred the horizontal opening: line 735

“Both groups of dogs avoided the circular opening and showed no preference for large opening over medium sized opening. They did however approach the bigger opening first. This was also true in mixed combinations, where openings differed in both shape and size. It is therefore possible that animals always approached the larger opening, but decided to investigate the second option and, as long as it was large enough (medium or large), they just walked through leading to no preference to attempt or walk through in those combinations. If this were the case, the first approach would be the most important measurement, while side first attempted and walked through would provide little information. In our data, there were only two trials when a subject approached a large opening, but walked through a medium sized one. Therefore, we can conclude that this is not the case and that side first attempted and walked through should also be taken into account. Untrained dogs had no preference between vertical and horizontal shape. This is in line with the previous results by Lenkei and her colleagues (experiment 3)^[1]^, where they showed that dogs did not rely on shape perception when arriving at the opening. Interestingly, agility trained dogs preferred the horizontal opening to the vertical one. This is probably due to most obstacles being horizontal or circular on the agility course, so through their training agility dogs learned how to handle these obstacles. The closest to a vertical opening that they encounter during training are weave poles, which are flexible and can be pushed aside, while the fences in our experiment were rigid and could not be easily moved. To investigate this further, more studies focusing on animals with different life experience and specific training are required. The agility trained dogs also had shorter latencies to reach the reward. Overall, the latencies were longer the smaller the barriers presented. The exact reason why the subjects avoided the circular opening remains unclear. In Experiment 2, both groups preferred the plant to the paper barrier, but the untrained dogs had a stronger preference than the agility trained dogs. The probability to walk through a barrier within the time limit was higher when the wooden (impassable) barrier was not present. Both groups attempted to walk through the correct side first more often in combination 2 (plant vs wood) when the difference in solidity of barriers was highest, while the preference was the weakest for combination 1 (plant vs paper) when the difference in solidity was the lowest.”

Point 11. Agility Training Effect in Experiment 2. The finding that untrained dogs outperformed agility-trained dogs in solidity-based choices is unexpected. While authors propose hypotheses (e.g., trained dogs' broader material experience reducing perceived solidity differences; social inhibition; conditioned avoidance of barrier destruction), this result demands cautious interpretation.

Recommendation for authors:
1. Highlight the counterintuitive nature: Emphasize this as an intriguing finding requiring cautious interpretation.
2. Propose targeted follow-ups: test dogs without owners present to evaluate "social inhibition" (though logistically complex); use less destructible barriers (e.g., push-flaps vs. tearable paper) to assess "avoidance of destruction" hypothesis.
3. Introduce attentional focus hypothesis: Suggest that agility training may prioritize processing trajectory/speed cues over detailed solidity assessment of static barriers—contrasting with untrained dogs' more exploratory approach.

**Our response**: We agree with the reviewer in all of these points. Maybe we were not clear enough but this finding was the main point discussed in the discussion of experiment 2. We already proposed the suggested follow-ups there. Furthermore, these results are referenced again in the general discussion, but focused more on the influence of training and there we again suggested that this effect should be investigated in future studies. We agree that these results require careful interpretation but we also recognized that further investigation of influence of training and experimental conditions is needed before we can draw a solid conclusion. We believe that this suggestion is already covered in the manuscript.

Minor Recommendations

Point 12. Keywords: Add "body size awareness" to the list. This term accurately reflects a core focus of the study and enhances discoverability.

**Our response**: We agree, but we are already at the limit of 10 key words. The word “body-size” is already in the name, while the terms self-awareness and body-awareness are mentioned as key words. Therefore, any search engine should connect this paper to “body size awareness”.

Point 13. Line 81: Standardize citation formatting to match the journal’s style guide. The current inconsistency deviates from the full citations used elsewhere.

**Our response**: corrected

1.	Capitain S, Cimarelli G, Blenkuš U, Range F, Marshall-Pescini S. Street-wise dog testing: Feasibility and reliability of a behavioural test battery for free-ranging dogs in their natural habitat. PLOS ONE 2024;19(3):e0296509.

2.	Nandi S, Chakraborty M, Lahiri A, Gope H, Bhaduri SK, Bhadra A. Free-ranging dogs quickly learn to recognize a rewarding person. Applied Animal Behaviour Science 2024;278:106360.

3.	Ward C. Greeting behavior between dogs in a dog park. 1 2020;(10):1–14.

4.	Range F, Ritter C, Virányi Z. Testing the myth: tolerant dogs and aggressive wolves. Proc Biol Sci 2015;282(1807):20150220.

5.	Cimarelli G, Marshall-Pescini S, Range F, Berghänel A, Virányi Z. Relationship quality affects social stress buffering in dogs and wolves. Animal Behaviour 2021;178:127–40.
