# Supplementary material for: Influence of agility training on body-size and object solidity perception in pet dogs
Source: PLoS One. 2026 Jan 7;21(1):e0338647. doi: 10.1371/journal.pone.0338647 (PMC12779069; doi:10.1371/journal.pone.0338647)
Supplement: Attachment — Submitted filename: Response to Reviewers2.docx [file pone.0338647.s013.docx]

Reviewer #3: Thank you for addressing most of my comments and for the corresponding revisions. A few points, however, remain insufficiently addressed. Below I clarify the rationale and offer specific suggestions for revision.
Point 5 — Demographic covariates
Thank you for the clarification. Here is why we ask you to account for sex and age in the models, even if these variables were not part of the a priori hypothesis.
• “We had no hypotheses about sex/age.” Adding covariates is not about testing a new hypothesis; it is a standard way to control potential confounding and to improve the precision of the main treatment estimate. Modern modeling guidance emphasizes that adjustment for relevant predictors reduces noise and yields more efficient standard errors (SEs) and confidence intervals (CIs). For age, please model the effect flexibly (e.g., restricted cubic splines) rather than as a strictly linear term.
See: Frank E. Harrell Jr., Regression Modeling Strategies.
• “Groups are similar.” Similar means at the group level do not preclude individual-level confounding, particularly when age variance is high; adjusting for informative covariates increases efficiency of the treatment effect even under randomization/balanced groups.
• “All dogs ≥14 months; body image is consolidated.” Physical maturity does not eliminate between-individual differences in attention, learning speed, and cognitive flexibility in adult dogs; age-related variation in these domains is well documented.
See: Wallis LJ, Range F, Müller CA, Serisier S, Huber L, Virányi Z (2014). Lifespan development of attentiveness in domestic dogs: drawing parallels with humans. Frontiers in Psychology, 5:71. https://doi.org/10.3389/fpsyg.2014.00071
• Empirical evidence for sex effects in canine cognition. Several studies report sex differences in dogs’ cognitive/spatial tasks.
See:
• Fugazza C, Mongillo P, Marinelli L. Sex differences in dogs’ social learning of spatial information. Animal Cognition (2017) 20(4):789–794.
• Mongillo P, Scandurra A, D’Aniello B, Marinelli L. Effect of sex and gonadectomy on dogs’ spatial performance. Applied Animal Behaviour Science (2017) 191:84–89.
• Junttila S, Huohvanainen S, Tiira K. Effect of sex and reproductive status on inhibitory control and social cognition in the domestic dog (Canis familiaris). Animals (2021) 11:2448.
• Reporting standards. ARRIVE 2.0 Explanation & Elaboration (Item 7, Statistical methods) explicitly states that nuisance variables should be considered in the analysis (e.g., as covariates); sex and age are listed among factors influencing outcomes. In addition, the NIH SABV policy expects sex to be factored into design, analysis, and reporting in vertebrate animal studies. Please either provide analyses with these covariates or offer a strong justification for excluding them.
See: Percie du Sert N. et al. (2020). Reporting animal research: Explanation and elaboration for the ARRIVE guidelines 2.0. PLOS Biology 18(7): e3000411.
Requested action:
— Refit all generalized linear mixed-effects models (GLMMs) and mixed-effects Cox models (CoxME) including sex and age as fixed covariates; model age with restricted cubic splines (3–5 knots). Report whether the agility-training effect persists and how SEs/CIs change.
— Provide a sensitivity analysis (models with vs. without these covariates); if feasible, exploratory training×age and training×sex interactions with appropriate caveats.
— Reflect these choices transparently in Statistical methods in line with ARRIVE 2.0 (E&E, Item 7).

Point 7 — Fatigue/motivation and trial-order effects
Thank you for the clarification. A subject random intercept adjusts baseline between-dog differences but does not model within-session trends (learning, fatigue, motivational decline). Such trends should be accounted for by an explicit trial index predictor; for inference, please treat trial as a continuous variable with a flexible functional form (e.g., restricted cubic splines) and, where possible, include a by-dog random slope for trial. Person-mean centering of the trial index (within-subject centering) is recommended to cleanly separate within- and between-dog components. This approach is standard for repeated-measures designs and avoids anti-conservative inference; splitting into thirds is unnecessary for the main model and can be reserved for descriptive checks. For time-to-success outcomes, include trial in Cox/CoxME; if warranted by the data structure, consider an extended Cox model with a time-varying covariate.
For Experiment 1: if each dog performed only one test, please acknowledge the limitation (no way to estimate a trial trend) and check batch/order at the session level; if multiple tasks/trials occurred per dog, include trial as above. Please provide sensitivity analyses (with vs. without trial) and partial-effect plots.
See: Barr DJ (2013). Random effects structure for testing interactions in linear mixed-effects models. Frontiers in Psychology 4:328.

**Our response:**

We agree that age and sex could be added as control variables. We also agree that the effect of age should not be simply modelled as linear. However, we were not sure if the restricted cubic splines (RCS) were the best method to model this effect. We would assume that younger dogs may be worse at assessing the physical parameters of their body due to lack of experience, while this ability may fade for older dogs. Therefore, the dogs that fall in between should outperform the rest. As for the effect of trial, we would actually expect it to be linear, as it is common for dogs to do 20 trials in an experiment, so we would expect some decline in motivation with trial but nothing major. With this in mind, introducing the age squared into the model may be a more accurate way to model the effect of age. Restricted cubic splines often have an issue of loss of interpretability and overfitting the data. To make sure we are using the best possible model for our data, we compared the Akaike Information Criterion (AIC) ^[1,2]^of models using squared term, RCS (4 knots) for age only, and RCS (4 knots) for both age and trial, see table below. The colour fill of the cells represents how well the model fits our data: green representing the best fit (lowest AIC), while the red represents the worst fit (highest AIC), and yellow and orange falling in between.

| Colour code |  |  |  |  |
| --- | --- | --- | --- | --- |
|  | Lowest AIC |  |  | Highest AIC |
| Comb12 | Original | Age squared; sex | Age splines (4); sex | Age and trial splines (4); sex |
| App | 140.2011 | 143.545 | 146.814 | 151.5677 |
| Att | 135.9755 | 137.5687 | 140.6845 | 146.1881 |
| Chosen | 138.8667 | 136.2802 | 138.164 | 142.5788 |
|  |  |  |  |  |
| Comb34 | Original | Age squared; sex | Age splines (4); sex | Age and trial splines (4); sex |
| App | 119.3054 | 122.2449 | 116.2574 | 119.6225 |
| Att | 99.10256 | 104.4944 | 103.5049 | 108.2469 |
| Chosen | 78.19002 | 83.19431 | 85.3913 | 89.85315 |
|  |  |  |  |  |
| Comb567 | Original | Age squared; sex | Age splines (4); sex | Age and trial splines (4); sex |
| App | 200.5669 | 204.4066 | 201.7396 | 208.3161 |
| Att | 131.7965 | 129.662 | 126.305 | 129.4156 |
| Chosen | 70.43967 | 117.9099 | 119.0174 | 123.829 |
|  |  |  |  |  |
| Comb8910 | Original | Age squared; sex | Age splines (4); sex | Age and trial splines (4); sex |
| App | 191.1069 | 194.8953 | 198.3063 | 201.556 |
| Att | 166.6473 | 172.1097 | 175.1665 | 179.193 |
| Chosen | 72.37238 | 137.4145 | 140.403 | 145.9742 |
|  |  |  |  |  |
| Solidity | Original | Age squared; sex | Age splines (4); sex | Age and trial splines (4); sex |
| App | 1269.479 | 1271.478 | 1275.186 | 1279.921 |
| Att | 991.5868 | 994.0419 | 997.616 | 990.9578 |
| Chosen | 351.5033 | 354.0858 | 356.6618 | 356.3391 |

Judging from the AIC comparison, both the model with the square term for age and with RCS for age would be good candidates. However, the square term models seem to have a better fit and paired with the negative sides of RCS, we decided to use the square term models. The models containing z-trial as a RCS yielded much higher AICs, so we decided to keep the z-trial as a linear variable as it both fits our expectations and seems to better fit our data. Additionally, it seems that RCS often overfitted our data. Here is an example of the comparison of effect of age in four different models (original - top left, squared - top right, RCS – bottom left, RCS for age and trial – bottom right).

It is clear that RCS overfitted the data simply because of the requirement to create the number of knots defined. This is usually useful when there are multiple age groups (e.g. in human studies), but for dogs we would only differentiate between young, middle-aged, and old dogs, so introducing more knots would only reduce the interpretability of the results.

We refitted all GLMM and Coxme models with added sex, z-transformed age, and z-transformed age squared. We updated the statistical analysis, results, and discussion(s) to fit the new models. We also explained in the statistical analysis the limitation of our design (each trial being different combination) and why we did not include z-trial for Experiment 1.
